# Bed Net Durability Assessments: Exploring a Composite Measure of Net Damage

**DOI:** 10.1371/journal.pone.0128499

**Published:** 2015-06-05

**Authors:** Jodi L. Vanden Eng, Adeline Chan, Ana Paula Abílio, Adam Wolkon, Gabriel Ponce de Leon, John Gimnig, Juliette Morgan

**Affiliations:** 1 Malaria Branch, Division of Parasitic Diseases and Malaria, Centers for Disease Control and Prevention, Atlanta, GA, United States of America; 2 Entomology Branch, Division of Parasitic Diseases and Malaria, Centers for Disease Control and Prevention, Atlanta, GA, United States of America; 3 Instituto Nacional da Saúde, Ministério da Saúde, Maputo, Mozambique; 4 United States President’s Malaria Initiative, Centers for Disease Control and Prevention, Atlanta, GA, United States of America; Swiss Tropical & Public Health Institute, SWITZERLAND

## Abstract

**Background:**

The durability of Long Lasting Insecticidal Nets (LLINs) in field conditions is of great importance for malaria prevention and control efforts; however, the physical integrity of the net fabric is not well understood making it challenging to determine overall effectiveness of nets as they age. The 2011 World Health Organization Pesticide Evaluation Scheme (WHOPES) guidelines provide a simple, standardized method using a proportional hole index (PHI) for assessing net damage with the intent to provide national malaria control programs with guidelines to assess the useful life of LLINS and estimate the rate of replacement.

**Methods:**

We evaluated the utility of the PHI measure using 409 LLINs collected over three years in Nampula Province, Mozambique following a mass distribution campaign in 2008. For each LLIN the diameter and distance from the bottom of the net were recorded for every hole. Holes were classified into four size categories and a PHI was calculated following WHOPES guidelines. We investigate how the size, shape, and location of holes influence the PHI. The areas of the WHOPES defined categories were compared to circular and elliptical areas based on approximate shape and actual measured axes of each hole and the PHI was compared to cumulative damaged surface area of the LLIN.

**Results:**

The damaged area of small, medium, large, and extra-large holes was overestimated using the WHOPES categories compared to elliptical areas using the actual measured axes. Similar results were found when comparing to circular areas except for extra-large holes which were underestimated. (Wilcoxon signed rank test of differences p< 0.0001 for all sizes). Approximating holes as circular overestimated hole surface area by 1.5 to 2 times or more. There was a significant difference in the mean number of holes < 0.5 cm by brand and there were more holes of all sizes on the bottom of nets than the top. For a range of hypothetical PHI thresholds used to designate a “failed LLIN”, roughly 75 to 80% of failed LLINs were detected by considering large and extra-large holes alone, but sensitivity varied by brand.

**Conclusions:**

Future studies may refine the PHI to better approximate overall damaged surface area. Furthermore, research is needed to identify whether or not appropriate PHI thresholds can be used to deem a net no longer protective. Once a cutoff is selected, simpler methods of determining the effective lifespan of LLINs can help guide replacement strategies for malaria control programs.

## Introduction

Recent escalation of national bed net distributions and universal coverage campaigns stimulated concern and investigation into the integrity and durability of long lasting insecticidal nets (LLINs) under ordinary use conditions. Mass distribution campaigns are recommended to deliver LLINs to households, rapidly increasing population coverage and improving equity of bed net ownership [1]. Attrition occurs following these campaigns, and LLINs degrade due to ordinary use and washing, compromising the physical integrity and chemical composition of the bed net. As a result, malaria control programs are challenged with devising LLIN replacement strategies that include considerations for net degradation and attrition in order to maintain high levels of protective coverage in the community.

In 2005, the World Health Organization Pesticide Evaluation Scheme (WHOPES) developed “Guidelines for laboratory and field testing of LLINS” [2]. This document focused on measuring the bioefficacy of LLINs. Methodologies to test the bioefficacy of LLINs have been thoroughly developed and standardized, including laboratory investigations using cone bioassay and tunnel tests [3] [4]. Moreover, following these guidelines, a variety of experimental hut studies have been performed to investigate the penetration through, feeding success, and mortality of mosquitoes among treated and untreated nets with and without holes [5–18].

In 2011, WHOPES published a second document “Guidelines for monitoring the durability of LLINs under operational conditions” [19]. In addition to the previously described measures of insecticidal activity (bioefficacy), these guidelines suggest two additional outcome measures to assess the useful life of a LLIN: a general measure of attrition and the physical condition or fabric integrity. However, until the recent mass distribution of LLINs, concern about the durability of bed nets in actual field conditions and investigation of the integrity and physical durability of the fabric have been lagging.

The integrity of different fabrics in laboratory settings have been compared using standard burst and tear strength tests identifying differences between weight, textures, and weave patterns [20]. Techniques to measure fabric integrity in the field; however, can be laborious and challenging. Precise laboratory measures of irregularly shaped holes are often impractical in general field conditions. Bed nets are often hanging in dark rooms where access may be a concern for privacy and where visibility is poor, making it difficult to detect holes. Some owners choose to take down the nets before allowing them to be evaluated which is not only time consuming, but also may risk the household neglecting to re-hang the nets after the evaluation is completed. Even with good visibility, it is possible to lose track of which holes were already counted on bed nets with a large quantity of holes. Moreover, there is often confusion on how to quantify and categorize different sized holes.

While challenges of accurately determining the counts, sizes, and locations of irregularly shaped holes on bed nets exist, there is an even greater need for a composite score or standard measure that quantifies the overall general physical condition of the net to address the question: “How do you determine when a net is no longer able to protect an individual sleeping under it from a malarious mosquito?” Until recently, there has been no consistent methodology for evaluating holes in nets that could serve as a standard by which net protectiveness could be measured. For example, is a net still protective with 2 small holes and one large hole, and how does that compare to a net with 4 medium sized holes? Prior to the development and routine use of the WHOPES recommendations, this question was addressed in many different ways with each study using unique approaches to measuring durability including different measurement tools, hole size categories, weights, algorithms, and thresholds. A few studies presented results of hole counts without a composite index of the physical durability [21–23]; however, most researchers developed a variety of simple algorithms to generalize the physical condition of the entire net. These include a weighted score [24–29], predetermined cutoffs such as ‘at least one hole’ [30–34] or ‘at least a certain number of holes of a specific size’ [35–42], size of largest hole [43], and sum of the perimeters of the holes[44]. Qualitative measures have also been suggested, for example Mutuku *et al*. reports that respondents perceived condition of the net may be more important than measuring the actual physical condition of the net [27]. These inconsistencies in assessing physical durability across studies not only make it nearly impossible to compare different net brands, years, and locations, but also make it difficult to conclude what constitutes a failed net.

Protection of an individual by a decaying net may decrease gradually, making it challenging to know exactly when a net is no longer functional and needs replacement. However, malaria control programs need to determine the optimal time to replace an entire population of nets. Many malaria control programs are replacing nets at three year intervals on the assumption that most nets will not be functional after that time. However, if the estimate of what is a “failed” net are too stringent, it means programs are replacing nets sooner than needed. If the estimate is too forgiving, then programs may be leaving people with nets that provide inadequate protection for an extended period of time. By incorporating information on the physical condition of LLINs in field situations and devising a method to better estimate a threshold of LLIN damage, programs may be better able to determine on average when a net fails and can design appropriate replacement strategies.

The 2011 WHOPES guidelines provide a standardized protocol to assess physical condition of LLINS in field situations. Included in this guidance is a composite measure of the nets’ physical condition called the proportional hole index (PHI) which is intended to provide an estimate of relative net damage for comparison among nets. This measure categorizes holes into four sizes. The number of holes of each size are counted and recorded during field evaluations. The PHI is calculated by multiplying the number of holes in each category by the corresponding weight (based on the estimated midpoint area of each hole category) and summing this across categories. Several studies have already reported bed net durability in field conditions using this methodology [26, 45–49] [50].

The PHI provides a much needed standardized approach to assessing the physical condition of a bed net in operational conditions. However, in order to develop a simple universal algorithm several generalizations are made on the size, shape, and location of holes. First, holes of all sizes are generalized into four categories based on an easy to measure finger/fist/head methodology. Holes > 25 cm in diameter are lumped together into one category, and holes < 0.5 cm are not counted. Second, the PHI assumes all holes to be circular when estimating the area of the holes. Third, the distribution of the holes of different sizes throughout the net is considered to be uniform. In other words, the PHI does not take into account the locations of the holes on the net. It is important to understand how these generalizations may influence the overall index and interpretation of the relative conditions of the nets. Moreover, the question remains, “at what PHI value is a net considered no longer protective?” Following the creation of the PHI, several studies have selected a cutoff (varying from 88 [27], 300 [26, 45, 46], to 768 [49]) as the hole index level at which a net is deemed no longer protective. Recent recommendations of the Vector Control Technical Expert Group at the Malaria Policy Advisory Committee Meeting (September 2013) proposed the following categories and PHI cut-offs to classify a LLIN: 0–64 good, 65–642 damaged, and 643+ too torn [51]; however, additional research is warranted.

This study investigates how the size, shape, and location of holes influence a composite hole index of overall net durability for two different brands of bed nets collected three years following a mass distribution campaign in Nampula Province, Mozambique. The present study uses data from a prospective evaluation of LLINs tagged during the mass LLIN distribution campaign in October 2008. The evaluation consisted of three annual cross-sectional surveys in either October or November of 2009, 2010, and 2011 and assessed the physical integrity of the LLINs following the WHOPES guidance as well as a more thorough laboratory measure[50]. In this paper we will explore the impact of these three generalizations (hole size, shape, and location) made using the current WHOPES PHI based on the availability of more detailed lab measurements.

## Methods

### Distribution and Evaluation

A prospective evaluation collected and measured the physical condition and insecticidal activity of LLINs tagged during a mass distribution campaign in Nampula Province, Mozambique in 2008. A thorough description of the study site, bed net distribution, and evaluation has been described elsewhere [50]. To summarize, a total of 4000 PermaNet 2.0 brand (polyester material, 100 denier, traverse knit structure [3.5.50]) and 2000 Olyset brand (polyethylene fibers, 150 denier, tulle knit structure [3.5.52]) nets were tagged at six different distribution sites. One month after the distribution, volunteers located 2023 (34%) of the tagged LLINs and created a sampling frame of households (N = 1549) owning these tagged LLINs by recording the geographic coordinates of each household using a global positioning system (GPS). The evaluation team returned to a randomly selected sample of households at 1, 2, and 3 years after the distribution to conduct follow up surveys and investigate the physical condition of the LLINs. The survey team performed a rapid assessment of the number and sizes of holes on the LLINs while in the field, and then collected the nets for more thorough evaluation in the laboratory. Overall, 447 LLINs (157 polyethylene and 290 polyester), 412 of those with at least one hole, were collected and evaluated throughout the follow up period.

### Hole Counting Procedures

The number and size of holes on the nets were measured during a rapid field assessment (for details see Morgan, *et al*. [50]) as well as more thoroughly in the laboratory. In the laboratory, nets were hanging in a natural (unstretched) position over a pre-constructed frame, and the size and location (distance from the bottom of the net to the center) of each hole were measured using a tape measure (in mm). During the first and second year, holes were measured along the major axis (longest section). As per WHOPES guidelines, holes < 0.5 cm were not counted. During the third year, the major and minor axes of the holes were measured. In addition, a count of the number of holes < 0.5 cm was recorded (including holes with a single torn thread in the weave). Although both field and lab data were collected, we present the more thorough data from the laboratory testing in this analysis.

### Data Analysis

During the measuring of holes in the laboratory, data were entered directly into handheld computers (Dell Axim X51, Round Rock, TX, USA) using questionnaires developed with Visual CE software (Syware, Inc., Cambridge, MA, USA). Data were downloaded directly into a MicroSoft Access database (Redmond, WA, USA). Data cleaning and analyses were performed using SAS version 9.32 (SAS Institute, Cary, NC, USA). Analyses were performed using SAS survey procedures (e.g. SurveyFreq, SurveyMeans) to adjust for clustering and the study design. Log transformations were performed in order to obtain geometric means for variables that were not normally distributed. When appropriate, domain analyses were performed to obtain results separately for each year and LLIN type; and were stratified by district. Proc Genmod was used to test for associations between hole size and location. When determining the proportion of holes at different locations of the net, it was noted that the total surface area of the roof was larger than the total surface area of the distance bands around the sides. Therefore the proportion was weighted to adjust for the difference in surface area of the roof panel. The nonparametric Wilcoxon signed rank test was used to compare paired measures of hole areas for the same nets such as the area based on PHI categories compared to areas using a circle or ellipse. The Wilcoxon-Mann-Whitney test was used to compare mean hole areas between two groups, Kruskal-Wallis test for more than two groups, and Spearman correlation to test for associations between ordinal variables. No adjustments were made for multiple testing.

### Hole Areas

**Fig 1:**
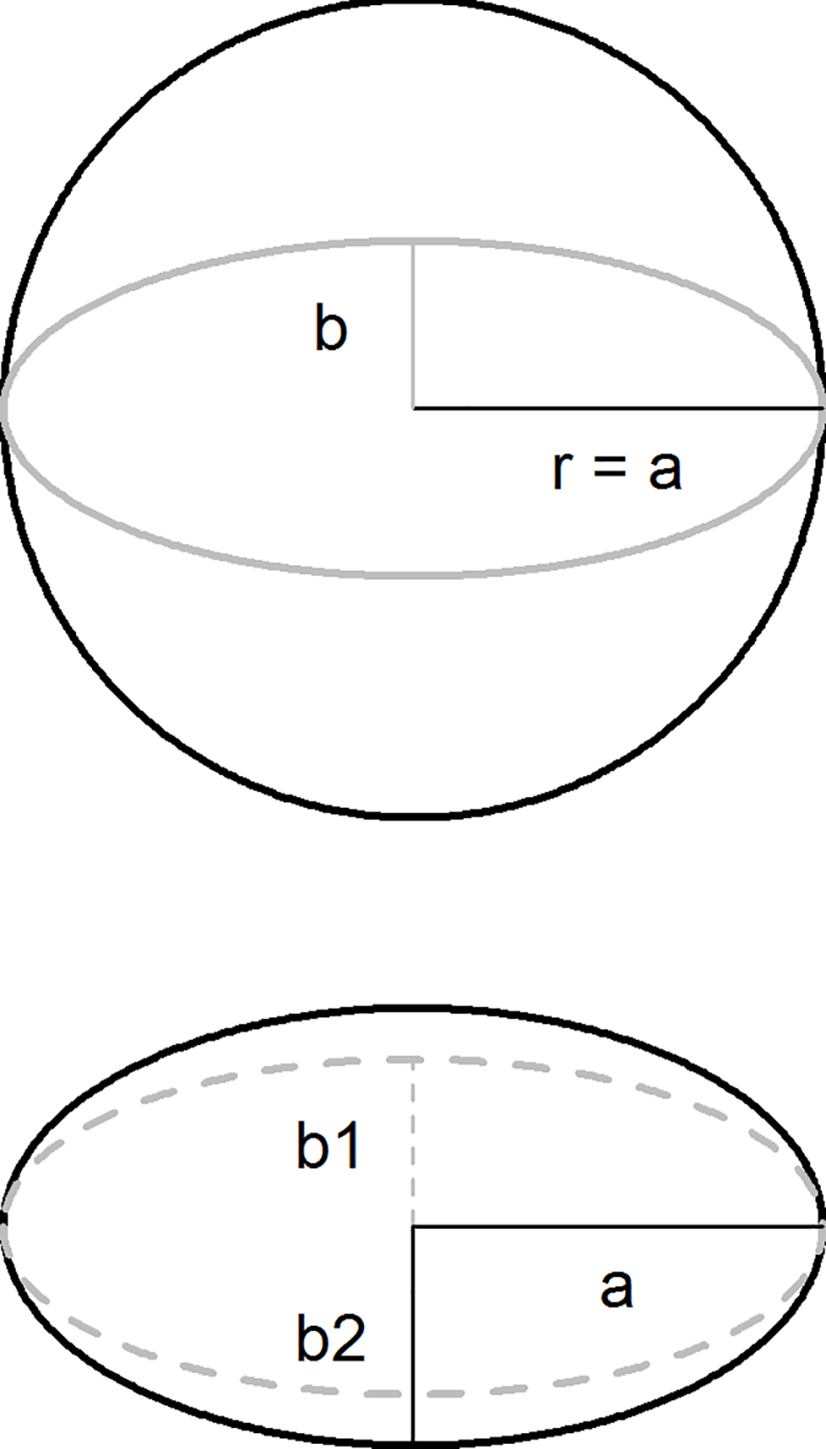
Diagram of hole measurements in the laboratory. A) Area of circle was calculated as A = pi * r^2^ where the radius, r, was calculated as half the length of the major axis. The area of an ellipse was calculated as A = pi*a*b where a is ½ the length of the major axis and b is ½ the length of the minor axis. B) Example of overall area of ellipse calculated using the ratio of major to minor axes (dashed ellipse = 2.4 the average ratio for polyethylene nets (b1 = half the length of the minor axis), solid ellipse = 1.9 the average ratio for polyester nets (b2 = half the length minor axis). Polyester nets: 100 denier, traverse [3.5.50], Polyethylene nets: 150 denier, tulle [3.5.52].

The PHI is based on a weighted sum of the different sized holes grouped into four categories. The weights are calculated with the assumption that the holes are circular (it uses the formula for an area of a circle to calculate the relative weight). This does not take into account the fact that many, if not most, holes on nets may be irregularly shaped. In the third year of the study, the major and minor axes of the holes were measured, and the ratio of the major to minor axes calculated. For perfectly circular holes, these values would be equal resulting in a ratio of one; however, for non-circular holes this ratio would be greater than one, and the larger the ratio, the more ‘narrow’ the hole (Fig 1).

Following conventional definitions, the major axis of a hole was defined as the length of the widest part of the hole and the minor axis as the length of the widest part that intersects perpendicular to the major axis. The area of a circle was calculated as A = pi*r^2^ where the radius, r, was calculated as half the length of the major axis (also known as the diameter). The area of an ellipse was calculated as A = pi*a*b where a is ½ the length of the major axis and b is ½ the length of the minor axis (Fig 1). Both the major and minor axes were measured in year three; however, only the major axis was measured in years one and two. In those years, the minor axis was calculated using a function based on a regression model of the major and minor axes from year three data adjusting for net type and WHOPES hole size category.

### Composite Index

The surface area of a net that is covered with holes was calculated using two different methods for both polyethylene and polyester nets at all three years: 1) by summing the areas of holes using the WHOPES categories and 2) by summing the area calculated from the actual measured diameters of each hole. The PHI was calculated as described previously[19] using the following four hole size groups: 1) smaller than a thumb (0.5–2cm), 2) larger than a thumb but smaller than a fist (2–10 cm), 3) larger than a fist but smaller than a head (10–25cm), and 4) larger than a head (> 25 cm). Holes equal to the cutoff (2, 10, and 25 cm), were included in the lower category. For the remainder of this paper these four categories will be referred to as small, medium, large, and extra-large holes, respectively. The number of holes of each size was counted and recorded. These counts were then multiplied by a corresponding weight for each specific size category and then summed across categories to get an overall hole index.

The index weights in the WHOPES guidance are based on the estimated mid-point area of each hole category relative to the area of the mid-point of the smallest hole category. More specifically, the formula for the area of a circle is used to calculate the hole area based on the mid-point radius of a category. The areas for the medium, large, and extra-large categories are then divided by the small hole area to get relative weights (see Table 1). For example, the midpoint of medium size holes (with diameters of 2 to 10 cm) is 6 cm. This gives a radius of 3 cm, and an area of 28.3 square cm. The weight is then calculated by dividing the area of the medium hole by the area of the small hole, 28.3/1.2 ~ 23 square cm. The midpoint for the extra-large hole category (> 25 cm) is assigned by WHOPES as 30 cm (this category has no upper bound). Note that holes of varying sizes within each category all get assigned the same area when calculating the PHI. For example, medium sized holes range from diameters of 2–10 cm, which corresponds to areas of 3.1 to 78.5 square cm; however, all medium holes regardless of size are assigned the same weight of 23. Consequently, the PHI is most accurate when hole sizes are either uniformly or symmetrically distributed within each category. For easier comparisons, a PHI based on the actual areas of the 4 categories is presented in this paper, rather than the PHI using the weights (e.g. the medium hole surface area was not divided by 1.2).

**Table 1:** Summary of WHOPES hole size categories, areas and corresponding weights.

Hole Size	Diameter (cm)	Midpoint	Range of Areas (cm^2^)	WHOPES Area (cm2)	WHOPES Weight
**Small**	0.5–2.0	1.25	0.2–3.1	1.2	1
**Medium**	2.0–10	6	3.1–78.5	28.3	23
**Large**	10–25	17.5	78.5–490.9	240.5	196
**X-large**	> 25	30*	490.9—max	706.9	578

* True midpoint is not known.

### Ethical Approval

Written consent was obtained by all study subjects or by their parent or guardian if they were under 16 years old. Participants signed or provided a thumbprint on two copies of the consent form, one of which was kept by the investigators and the other was given to the participants. Approval for this study and consent procedure was obtained from the Bioethical Committee of the Ministry of Health, Mozambique (CNBS, Maputo, Mozambique). CDC investigators participated under a non-research determination on the basis of routine public health evaluation.

## Results

### Bed net holes summary

Results of the LLIN durability study using the WHOPES methodology can be found in Morgan *et al*. [50]. To summarize, 447 bed nets were collected in the field and 445 were available for hole counting in the laboratory. Over the three years of follow up, 412 bed nets were found to have holes: 50, 58, and 47 polyethylene and 73, 97, and 87 polyester nets in 2009, 2010, and 2011 respectively. Of the remaining 33 nets without any holes 1, 1, and 0 were polyethylene, and 24, 7, and 0 polyester nets in 2009, 2010, and 2011 respectively. Three LLINs were considered outliers and removed from the analysis because they were severely damaged and would likely have skewed the results (2 polyethylene from year two with 1185 and 2634 holes, and 1 polyester LLIN from year three with 3861 holes. A total of 17,320 holes larger than 0.5 cm were counted on the nets with 63.4% of the holes size 0.5–2 cm, 30.4% size 2–10 cm, 4.5% size 10–25 cm and the remaining 1.8% greater than 25 cm in diameter. The mean number of holes per net increased over time from an average of 28.3 ansd 13.2 in 2009 to 87.7 and 43.0 in 2011 for polyethylene and polyester nets, respectively.

### Generalization # 1: Size of holes

#### Classification of holes into four categories

**Fig 2:**
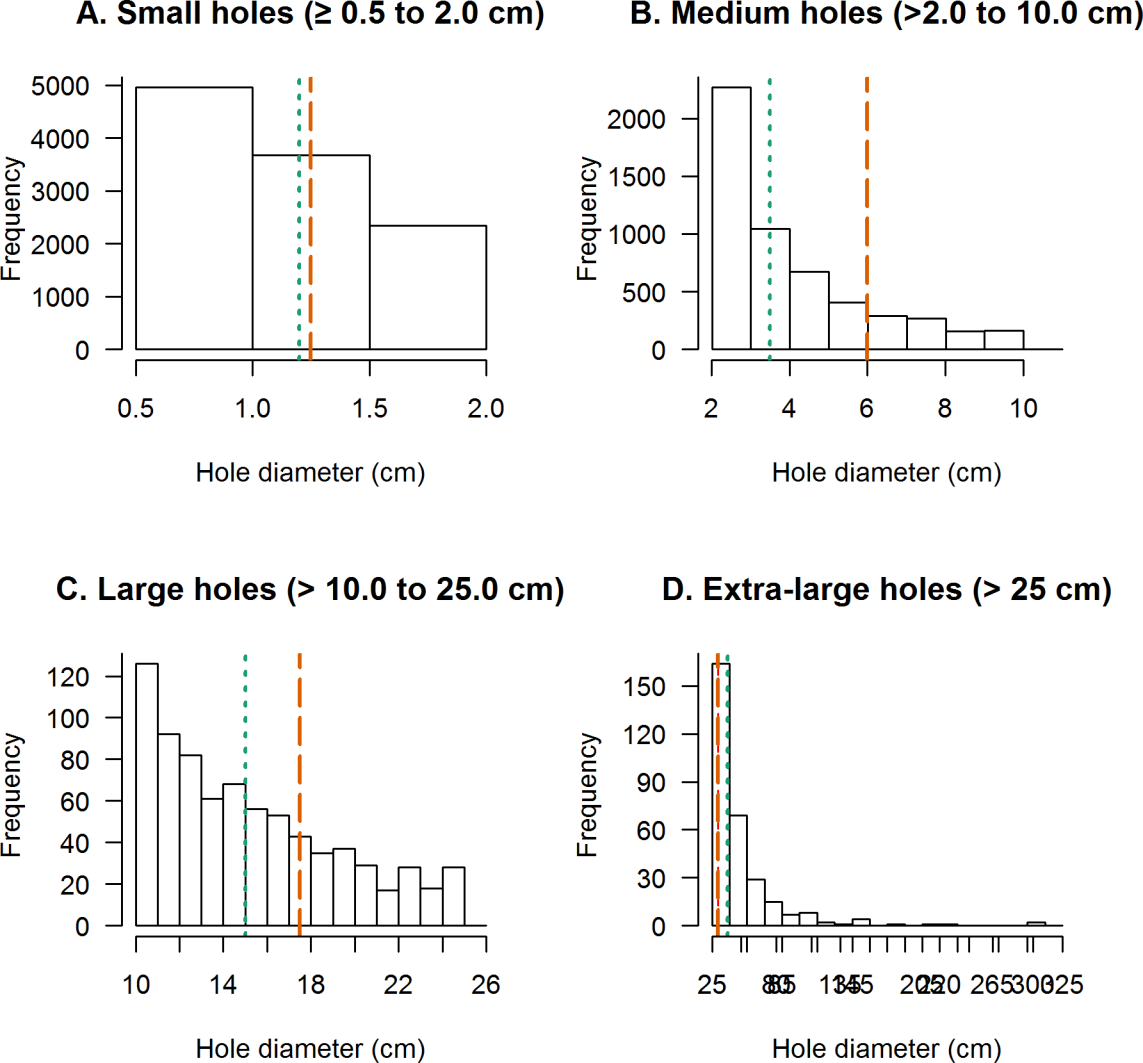
Histogram of measured hole diameters for each assigned WHOPES hole category. The midpoint hole diameter used for the WHOPES category weight is indicated by an orange dashed vertical line. The median hole diameter in each category is indicated by a green dotted vertical line.

In general, there were many more small holes than large holes. The number of holes in each of the WHOPES hole categories overall were as follows: 57.2% of all holes were small, 34.7% medium, 5.7% large, and 2.5% were extra-large respectively in polyethylene nets and 70.6%, 25.4%, 3.0%, 0.9% were small, medium, large and extra-large holes in polyester (Table 2). The distribution of holes within each size category followed the same decreasing trend in all four hole size categories; there are fewer larger sized holes than smaller sized holes. Fig 2 presents the distribution of the hole diameters in each hole size category. The dashed vertical lines indicate the diameter midpoint used for calculating the WHOPES areas for each of the four categories. Areas for holes with smaller diameters than this midpoint were overestimated, whereas areas for holes with larger diameters were underestimated.

**Table 2:** Calculated hole areas (cm^2^) for each WHOPES size category, by net type.

						Part A: Area of a circle* (cm^2^)			Part B: Area of an ellipse* (cm^2^)		
Net Type	WHOPES Hole Category	# Nets with holes	# Holes	% of all hole	WHOPES area (cm^2^)	Median	Geometric Mean	95% CI	Median	Geometric Mean	95% CI
**Polyethylene**	Small	152	5326	57.2	1.2	1.3	1.3	(1.3–1.4)	0.8	1.0	(1.0–1.1)
	Medium	151	3228	34.7	28.3	9.5	12.1	(11.6–12.5)	4.1	5.3	(5.1–5.5)
	Large	106	530	5.7	240.5	173.0	184.9	(176.2–194.0)	49.0	50.9	(48.0–54.0)
	Extra-large	72	229	2.5	706.9	1189.5	1533.6	(1346.4–1746.9)	246.7	328.9	(278.0–389.1)
**Polyester**	Small	252	5655	70.6	1.2	0.8	1.0	(0.9–1.0)	0.7	0.8	(0.76–0.83)
	Medium	217	2034	25.4	28.3	9.3	11.4	(10.8–11.9)	4.7	5.9	(5.6–6.2)
	Large	105	243	3.0	240.5	141.0	166.3	(154.0–179.5)	51.8	67.2	(60.3–74.8)
	Extra-large	46	75	0.9	706.9	1046.5	1316.6	(1073.5–1614.7)	132.6	347.2	(250.7–480.8)

Two different methods for calculating the area were used: A) formula for area of a circle based on measured major axis of each hole, B) formula for area of an ellipse based on length of major and minor axis (for years 1 and 2, minor axis was approximated using a ratio of major to minor axes). 95%CI = 95% confidence interval for the geometric mean.

*Wilcoxon sign rank test of difference between WHOPES area and area of a circle or area of an ellipse, all comparisons at each size p-values < 0.0001. Polyester nets: 100 denier, traverse [3.5.50], Polyethylene nets: 150 denier, tulle [3.5.52].

The geometric mean and median areas calculated using actual axes measurements based on the area of a circle were in general smaller than the area using the assigned WHOPES midpoint for holes in the medium and large categories (Wilcoxon sign rank p-value < 0.0001 for all comparisons; Table 2, Part A). The difference was especially pronounced in the large hole category which had a mean hole area of 184.9 cm^2^ for polyethylene and 166.3cm^2^ for polyester nets compared to 240.5 cm^2^ based on the WHOPES midpoint.

Extra-large holes are classified as any hole with a diameter > 25 cm, and the WHOPES index uses a midpoint diameter of 30 cm to calculate the area. In this hole category, close to ¾ of the holes meet or exceed the midpoint (75.5% polyethylene and 76.0% polyester) and as a result their areas may be slightly to grossly underestimated using the WHOPES midpoint (Fig 2D, Table 2, Part A). In the extra-large hole category polyethylene nets had a median hole area of 1189.5 cm^2^ and polyester nets had 1046.5 cm^2^ compared to 706.9 cm^2^ based on the WHOPES midpoint (p-value <0.0001 for both).

#### Impact on Composite Index

**Fig 3:**
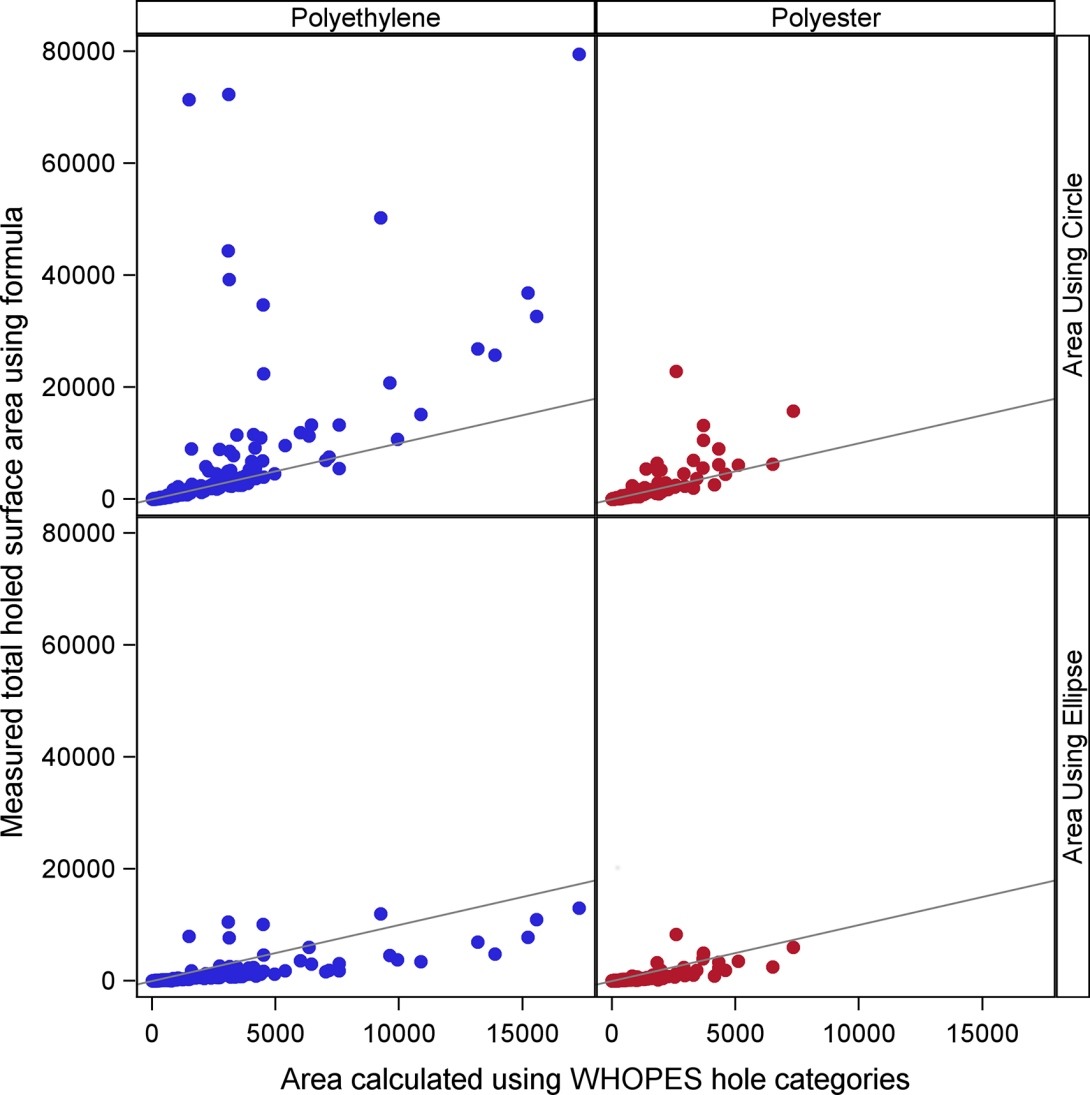
Scatterplot of surface area calculated using WHOPES Proportional Hole Index categories vs measured total holed surface area of net using A) formula of a circle, and B) formula of an ellipse by net type. Each point represents one net. The black lines represent the diagonals. Polyester nets: 100 denier, traverse [3.5.50], Polyethylene nets: 150 denier, tulle [3.5.52].

Overall, the median and geometric mean of the composite interrupted surface area was larger using the WHOPES PHI categories than the actual estimated circular areas for polyester nets across all three years and for polyethylene nets in 2009; whereas the median and mean tended to be smaller for polyethylene nets in 2011 (Table 3 Part A, Fig 3, row 1). Significant differences in calculated areas based on PHI categories or estimated circular areas were seen for polyethylene nets in 2009 and 2011 and polyester nets in 2009 and 2010 and nominally in 2011. No statistically significant difference was found for polyethylene nets in 2010 using nonparametric tests.

**Table 3:** Average total net surface area covered with holes by net type and year among nets with holes.

				Area Using WHOPES PHI Categories (cm^2^)			Part A: Area Using Circle (cm^2^)				Part B: Area Using Ellipse (cm^2^)			
Net Type	Year	# Nets	# Intact nets	Median	Geometric Mean	95% CI	Median	Geometric Mean	95% CI	p-value*	Median	Geometric Mean	95% CI	p-value*
**Polyethylene**	2009	50	1	442	394	(264–588)	261	306	(191–501)	0.02	96	112	(75–167)	<0.0001
	2010	56	1	1546	1167	(763–1786)	1723	1179	(707–1966)	0.94	442	368	(238–569)	<0.0001
	2011	47	0	3078	2614	(1873–3649)	4112	3857	(2275–6538)	0.0007	1376	1089	(677–1749)	<0.0001
	Total	153	2	1429	1049	(810–1358)	1256	1096	(784–1532)	0.32	380	348	(260–466)	<0.0001
**Polyester**	2009	73	24	65	53	(33–86)	41	34	(20–58)	<0.0001	22	20	(13–32)	<0.0001
	2010	97	7	273	209	(146–299)	159	144	(97–215)	<0.0001	82	45	(52–106)	<0.0001
	2011	86	0	585	504	(377–673)	390	398	(274–577)	0.06	185	172	(122–241)	<0.0001
	Total	256	31	261	190	(149–243)	138	134	(102–177)	<0.0001	80	68	(53–87)	<0.0001

Calculated using WHOPES categories (small = 1.23, medium = 28.3, large = 240.5, extra-large = 706.9), Part A) area assuming circular shaped holes, and Part B) area assuming elliptical shaped holes.

* p-value = Wilcoxon signed rank test comparing total hole area based on WHOPES PHI categories to areas based on formulas of a circle and ellipse (by net type and year). 95% CI = 95% confidence intervals for the mean. Polyester nets: 100 denier, traverse [3.5.50], Polyethylene nets: 150 denier, tulle [3.5.52].

The geometric mean estimate of holed surface area was roughly five times greater for polyethylene than polyester nets (all years Wilcoxon-Mann-Whitney Z = 8.4, p-value < 0.0001) and there was a difference in holed surface area by year for each brand (Kruskal-Wallis Chi-Square = 45.4 polyester, 37.1 polyethylene, p-values <0.0001 for both brands).

#### Extra small holes (< 0.5 cm)

The laboratory hole counting procedure was modified in 2011 to include a tally of extra-small sized holes (< 0.5 cm in size) on all collected nets. Three nets, one polyester and two polyethylene, were not evaluated for extra-small holes. In total 16,096 holes of this size were tallied over the 130 nets evaluated (Table 4). One net with 9972 of these holes was deemed an outlier and was not included in further analysis. There were relatively similar number of the holes on different sides of the net; however the top of the net had fewer holes than the sides. Polyester nets brand nets tended to have more small holes than polyethylene (median 44.5 vs 8.3, p-value < 0.0001).

**Table 4:** Characteristics of nets with extra-small holes (< 0.5 cm diameter) for 130 nets in the third year 2011.*

			Number of Extra-Small Holes Per Net					
Net Type	Total Number of Nets	Total Number of Extra-Small Holes	Q1	Median	Q3	Geometric Mean	95% CI*	p-value**
**Polyethylene**	45	792	2.6	8.3	20.8	8.9	(6.1–13.0)	<0.0001
**Polyester**	85	5332	19.8	44.5	84.5	39.6	(31.9–49.1)	
**Overall**	130	6124	10.3	27.0	61.5	23.6	(19.4–28.7)	

*95% CI = 95% confidence interval for the geometric mean

** p-value = Kruskal-Wallis test. Polyester nets: 100 denier, traverse [3.5.50], Polyethylene nets: 150 denier, tulle [3.5.52].

**Fig 4:**
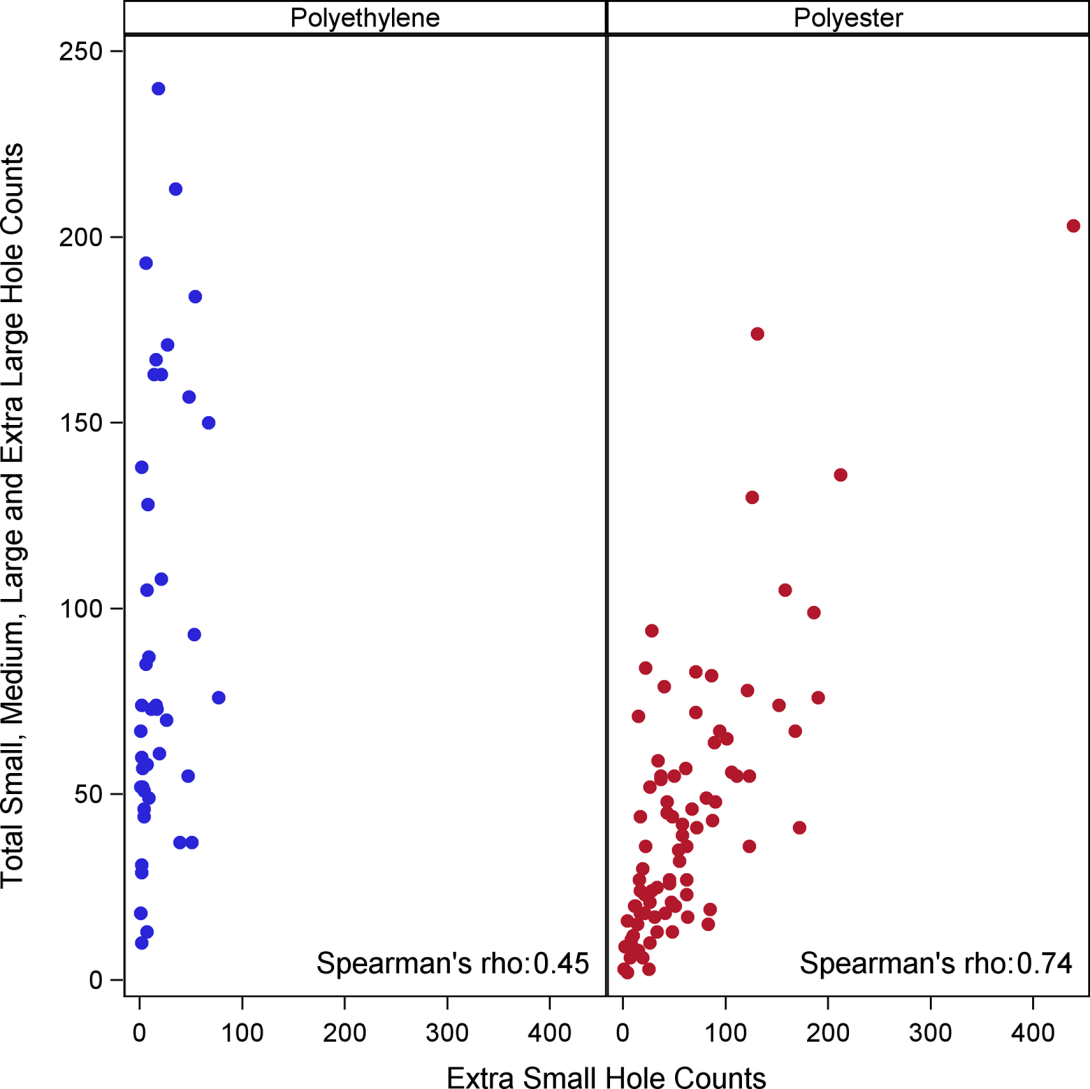
Scatterplots of number of extra-small size holes (< 0.5 cm) vs total number of small, medium, large and extra-large size holes per net by type for 130 nets during the 3^rd^ year of study (2011). Each point represents one net. Polyester nets: 100 denier, traverse [3.5.50], Polyethylene nets: 150 denier, tulle [3.5.52].

To a certain extent, the number of extra-small holes in a net corresponded to the number of all other sized holes (Fig 4). The level of correlation between the number of extra-small holes and number of all other holes differs between polyethylene and polyester nets brands (Spearman’s rho = 0.45 and 0.72, respectively). The number of extra-small holes for polyester has a stronger correlation to the number of other sized holes on the net, whereas polyethylene nets have a smaller correlation. Polyethylene nets seem to have fewer number of extra-small holes overall, but they have more holes of larger sizes.

### Generalization #2: Shape of holes

#### Ratio of major to minor axes

In the third year of the study, the major and minor axes of the holes were measured, and the ratio of the major to minor axes calculated. Small holes had a major axis about twice as long as the minor axis, and larger hole sizes were less circular (narrower) as can be seen by the increasing ratios with increasing size (Table 5, Fig 1). In general, polyester nets tended to have more circular holes compared to polyethylene in all but the largest hole size category. Overall, the major axis was approximately twice as long as the minor axis (ratio of major to minor axes: 2.44 for polyethylene and 1.87 for polyester) suggesting the holes may be poorly approximated by a circle. Ratios equal to or greater than two indicate the circular area approximation is about twice as large as or larger than the estimate using the area of an ellipse, resulting in an overestimate of the damaged area and an inflation of the PHI.

**Table 5:** Ratio of length of major axis to the minor axis of a hole by net brand and WHOPES hole size categories.

			Ratio	
Net Type	WHOPES Hole Category	# Holes	Mean	95% CI
**Polyethylene**	Small	2203	1.85	(1.81–1.89)
	Medium	1504	2.60	(2.52–2.68)
	Large	273	4.17	(3.66–4.68)
	Extra-large	139	6.57	(5.21–7.93)
	Overall	4119	2.44	(2.37–2.51)
**Polyester**	Small	2488	1.57	(1.52–1.62)
	Medium	1043	2.16	(2.08–2.23)
	Large	131	2.98	(2.57–3.40)
	Extra-large	34	10.59	(5.40–15.78)
	Overall	3696	1.87	(1.80–1.94)

95% CI = 95% confidence interval for the mean. Polyester nets: 100 denier, traverse [3.5.50], Polyethylene nets: 150 denier, tulle [3.5.52].

#### Hole area using ellipse

The geometric mean and median areas for holes in the small, medium, and large categories calculated based on actual axes measurements using the formula for the area of a ellipse were smaller than areas calculated using either the area of a circle or WHOPES hole categories (Wilcoxon sign rank p-value < 0.0001 for all comparisons, Table 2, Part B).

The total surface area of a net that is covered with holes was also calculated by summing the elliptical areas calculated from the actual measured diameters. The geometric mean and median area calculated assuming the holes were elliptical were much smaller than those calculated using either a circular area or the WHOPES categories (Wilcoxon sign rank p-value < 0.0001 for all comparisons). The total hole areas were greater for polyethylene than polyester nets (all years Wilcoxon-Mann-Whitney Z = 7.6, p-value < 0.0001; Table 3 Part B, Fig 3, row 2).

### Generalization #3: Location of holes

**Fig 5:**
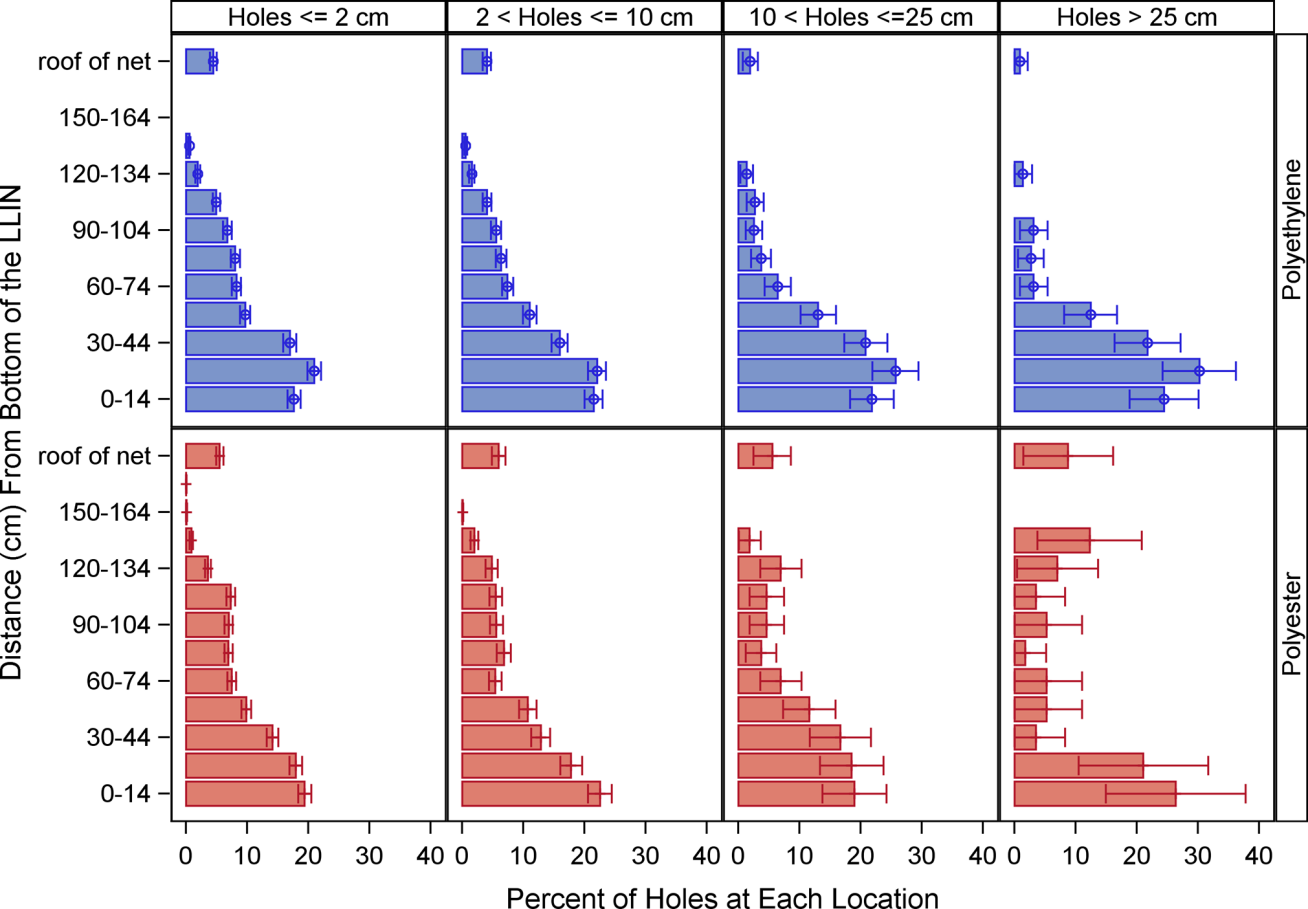
Percent of holes of each WHOPES size category located at varying distances from the bottom of the net by type. Error bars represent 95% confidence limits. Polyester nets: 100 denier, traverse [3.5.50], Polyethylene nets: 150 denier, tulle [3.5.52].

Most holes of any size were located in the lower quarter of the LLIN for both brands (less than 37.5 cm from the bottom for polyester, and 45cm for polyethylene) (Fig 5). The roof of the net also had some holes, with a larger proportion of holes seen on the roof of polyester than polyethylene nets after adjusting for differences in surface area. In general the pattern of distribution of holes by location on a net appear similar by hole size and net brand with an increasing proportion of holes closer to the bottom of the net. The size of the hole was significantly associated with the percent of holes at each location for polyethelyene (p < 0.0001) but not for polyester nets (p = 0.76).

### Influence of hole sizes on the PHI

If a specific threshold value of hole surface area is selected as part of a set of criteria for replacement of nets such as the cut-offs proposed by the Vector Control Technical Expert Group, the corresponding number of holes of each size required to meet the threshold can be extrapolated. As an example, consider a cutoff of PHI larger than 299 for an unusable net [24, 26, 45, 46], then identifying just one extra-large hole (PHI of 398) in a net would deem it no longer protective. Likewise, two large holes would have a PHI of 392 and also deem the net no longer protective.

Depending on the chosen cutoff, many nets may be determined to be unusable just by evaluating the number of extra-large and large holes on the net. Using cutoffs of PHI = 1000, 750, 500, 300, and 100 with the 452 nets in this study, resulted in 27.7, 33.8, 41.9, 51.8, and 67.1 percent of nets deemed unusable, respectively. Of these, 74.0, 79.3, 80.6, 76.5, and 78.2 percent of the nets exceeded the cutoff just by counting the extra-large and large holes alone (see Table 6 for results by net type). Therefore, roughly 75–80% of the nets considered no longer protective using different PHI cutoffs could have been detected by only considering holes larger than a fist; however, results vary by net type.

**Table 6:** Proportion of nets determined to be unusable by 1) counting all holes on the net, 2) counting only number of extra-large (XL) and large (L) holes on the net for different PHI cutoffs by net type.

		Proportion of LLINS not 'serviceable'				
		Counting all holes on net		Based on counting L or XL holes alone		
**Net Type**	**PHI Cutoff**	**n**	**%**	**n**	**%**	**Sensitivity**
**Polyethylene**	1000	84	53.5	70	44.6	83.3
	750	92	58.6	80	51.0	87.0
	500	109	69.4	92	58.6	84.4
	300	119	75.8	105	66.9	88.2
	100	135	86.0	118	75.2	87.4
**Polyester**	1000	42	14.5	24	8.3	57.1
	750	61	21.0	42	14.5	68.9
	500	80	27.6	61	21.0	76.3
	300	114	39.3	74	25.5	64.9
	100	166	57.2	118	40.7	71.1

Sensitivity reflects the percentage of unusable nets detected by counting the XL and L holes alone out of all unusable’ nets determined by counting all holes. NOTE: these results are for all 447 nets (including those with no holes).

## Discussion

The physical durability and usable lifetime of a bed net is of programmatic interest in order to determine the most cost effective timing and distribution of replacement nets. According to WHOPES an LLIN is a product that retains biological efficacy for at least 3 years, defined as 80% of nets with either ≥ 95% knock-down or ≥ 80% mortality [52]. This definition is entirely based on the measurement of insecticidal activity in the nets after general washing and use but does not contain criteria for some measure of fabric integrity including burst strength. Evidence from recent investigations following LLIN mass distribution campaigns suggests that the actual lifetime of the nets in the field is shorter than expected [48].

The 2011 WHOPES guidelines provide a much needed standardized approach to assessing the practical lifetime of a bed net in operational conditions. They not only help malaria control programs devise timely replacement strategies, but also provide programs with a quantitative approach to compare different fabrics and determine the most appropriate and cost effective bed nets for their specific situation. However, the proportional hole index makes several generalizations related to size, shape, and location of holes that limit the utility for both of these objectives. Public health practitioners may want to consider additional methods to help optimize the use of the PHI for specific program goals when applying the WHOPES assessment in the field.

When the primary program goal is only to determine whether or not a group of nets are in good condition or need replacement, it may not be necessary to count all of the holes in every net. Instead one could just count holes up until a specific threshold is met. Results of this study found that roughly 75–80% of the nets exceeding a PHI cutoff could be identified just by counting holes larger than a fist which are easy to identify in field conditions. Depending on the designated threshold for determining a net no longer protective, a simple guide or decision tree may be created to facilitate bed net assessments and reduce the number of holes counted per net as well as the number of nets that need all of their holes counted. The simplicity of this method may make it suitable to be incorporated in large household surveys such as a malaria indicator survey. In addition, this type of simple hole counting method could be used for study designs such as lot quality assurance sampling as part of a program’s monitoring strategy for bed net replacement.

Even if a simplified method such as a decision tree or a more thorough method using a PHI cutoff is promoted, the range of hole areas within the simple WHOPES categories may lead to some inconsistencies. For example, a cutoff that is based on 5 or more large holes (or a PHI of 800) may have unintended consequences. It is possible that a net with 4 large 24 cm holes (~ 1808.6 square cm, PHI = 784) would be classified as still functional, whereas a different net with 5 large 10 cm holes (392.5 square cm, PHI = 980) would be classified as needing replacement even though the total damaged area is more than four times larger in the former net. Since the actual size of the hole is not recorded, just the number of holes in each category, this inconsistency would be largely undetected.

Moreover, an important question of interest “what cut-off(s) should be used to determine when a bed net or LLIN population has reached the ‘end of useful life’?” remains outstanding. The recent recommendations of the Vector Control Technical Expert Group at the Malaria Policy Advisory Committee Meeting proposed PHI cut-offs based on existing evidence to classify a LLIN into the following categories: 0–64 good, 65–642 damaged, and 643+ too torn [51]. However, gaps in knowledge still exist in this area and many unresolved issues about what hole sizes, shapes, and locations are most important for mosquito penetration and malaria transmission have yet to be answered.

When the primary program goal has a more research focused objective to develop a system for evaluating existing bed nets to determine which model may most suitable in a particular area a more thorough investigation of the net condition may be necessary. This objective often requires a more rigorous research approach performed on a limited number of bed nets to understand the formation of holes; their size, shape, location, and cause of damage as well as estimate the total damaged areas of bed nets over their lifetime. In these cases improvements on the PHI may be warranted to get a more accurate picture of bed net damage, as well as to properly assess a new model, weave, or user setting and location and especially when new brands are introduced into the market.

Results from this study show hole sizes were not symmetrically distributed within hole categories, but decreased in number as the size increased. As a result, the PHI overestimated the amount of holed surface area for small, medium and large holes. In contrast, extra-large holes were found to be underestimated compared to circular estimates. When considering the PHI composite measure, it was found to overestimate area for all years when compared to an elliptical based measurement. In addition it overestimated the area in polyester nets in all three years when compared to circular estimates using actual measured axes. However, the PHI underestimated the actual measured area in polyethylene nets for 2010 and 2011 compared to circular estimates, indicating that underweighting by using the midpoint from the extra-large hole categories has a greater impact (though fewer holes) than the overweighting from the small, medium, and large categories. This finding was more prominent in polyethylene than polyester nets as they tended to have more extra-large holes.

Extra-small holes (less than ½ cm) are excluded in the WHOPES guidelines. They are likely too small for easy passage of mosquitos and therefore when considering efficacy of an LLIN, the net would still be considered an effective barrier for its user even with an extra-small hole. However, when explicitly considering the physical durability of LLINs in the context of lifespan and replacement, extra-small holes suggest overall wear on the LLIN, can unravel leading to larger holes, and may serve as an indicator of a reduced lifespan of the net compared to one without extra-small holes. Extra-small holes are often very challenging to observe and measure during field studies [53], as such, it may be beneficial to use a proxy to estimate their numbers such as the number of other sized holes on a net. A plot of the relative proportion of the number of extra small holes on a net compared to the number of all other sized holes on a net revealed a difference between brands. While polyester nets show an increasing number of extra-small holes with an increasing number of all other sized holes, polyethylene nets have fewer extra small holes relative to increasing numbers of larger sized holes. There are many potential explanations for this finding. One such explanation would be that due to weave patterns, a small hole in polyethylene may more rapidly expand to become a larger hole, so the likelihood of observing a small hole on polyethylene nets is reduced.

The formula for the PHI assumes holes are circular; and in the field a hole is categorized based on whether one can insert a finger, fist, or head through the hole. These measures overlook the possibility of irregularly shaped holes. This study found that most holes were 2 to 10 times longer in one direction (more long and narrow than circular), and as result the area assuming the hole is circular tends to overestimate the actual holed area by roughly 2 to 10 times. It is possible that the formula of an ellipse better approximates the true holed area; however, this too does not account for irregularly shaped holes and is laborious to obtain.

The largest proportion of holes was found to be located at the bottom 1/4 of the net. Certain sections of a net may be more prone to tears based on use or other factors; also certain areas of the net may be more likely for mosquitos to penetrate than others. By understanding what parts of nets are most vulnerable, measures can be taken to strengthen nets at these locations. Future studies may want to take into account not only the size of the hole, but also the location when calculating a composite index scores by weighting or adjusting the score based on the location of the hole (e.g. weight holes on the top of a net more than those on the bottom of the net if future studies show mosquitos are more likely to enter holes in the top of the net). Also note that the PHI ignores repairs in nets such as stitches, knots, patches.

There are some limitations of the current study that should be noted. This study was initiated prior to the initial release of the WHOPES guidance and as a result several of the logistical methods did not follow the finger/fist/head recommendations exactly. However, this analysis consisted of the more precise lab measurements, and should not be affected by this deviation in the design. Lab recorders were requested to measure the longest section of a hole (major axis), and for the 3^rd^ year also the perpendicular to the major axis (minor axis). It is possible that for irregularly shaped holes some measurement error was introduced. In addition, the shape of holes varied greatly, and approximating the holes as a circle or ellipse has potential for error. This study took place prospectively over the course of three years, during which 16.6% of the households did not retain all of the bed nets received during the campaign. A survivorship bias may have been introduced into this data if the condition of the nets lost due to attrition differed from that of nets measured in this evaluation. In addition, the extra-small hole analysis only took place on nets during the 3^rd^ year of data collection, and results may not be representative for younger nets.

This paper demonstrates that measuring the physical integrity of bed nets is multifactorial and requires a thorough understanding of the size, shape and location of holes. More research is needed to potentially refine the PHI measurement tool as well as improve the manufacture and development of more durable bed nets. Moreover, physical integrity is not the only determinant of overall protectiveness of a bed net and it is important to include insecticide activity and bioefficacy in an assessment. A few experimental hut studies have looked at the impact of insecticide on bed nets with and without holes [6, 7] [10, 54] [11–13, 15–17]; however, the impact of holes of different size, shapes and locations is not clear. More research is needed to evaluate both bioefficacy and physical integrity for LLINs.

## Conclusions

There is a programmatic need to develop a standard, reproducible, logistically simple, and comparable method to assess net condition under normal use environments. The PHI provides a standardized approach to estimate the total surface area (or relative area using the weights) damaged by holes on a net. Results of the current detailed analysis using bed nets collected over three years in Mozambique reveal some of the potential weaknesses of the PHI including: underestimating the contribution of really large holes, overestimating the contribution of smaller holes, ignoring extra small holes and hole location, and oversimplifying the hole shape as circular. More research is needed to better refine the PHI and to understand the factors associated with hole initiation and enlargement in field conditions. Information about the size, shape, location and type of holes may help develop a simple field strategy for assessing net usability in terms of physical integrity that will help programs develop plans for repair or replacement of nets. More importantly, further investigation is needed to determine what hole size(s) put net users at the greatest risk in order to determine the optimal time for net replacement.

Bed net monitoring and evaluation (M&E) activities including coverage surveys and malaria indicator surveys are an integral part of national malaria control program activities and provide information on general bed net ownership and use. The PHI is designed to help determine whether a large population of bed nets are still protective or should be replaced; however, in field situations identifying and counting all of the holes in a net, tabulating the data, and calculating a PHI may be laborious and time consuming. Due to the large volume of nets under evaluation, a simpler method of determining the usability of a net based on its physical condition that could be easily incorporated in existing M&E activities would be of great value.

Physical integrity assessments including both in depth analysis of a small sample of nets and simple field counting strategies on larger numbers of nets during household surveys can be used in conjunction with insecticidal activity monitoring to enhance malaria control efforts in the field and can help guide manufacturers to build longer-lasting bed nets.

## Supporting Information

S1 Table Net Source Data. Columns are as follows: NetID = unique Net ID, NetType = Brand of net, Year = year net was assessed, and total CNT = total numbers of damaged areas on net including holes, knots, stitches and seam tears.
(TXT)

S2 Table Net Holes Source Data. Columns are as follows:: NetID = unique Net ID, DamageID = unique ID for point of net damage, Damagetype = type of net damage (1 = hole, 2 = stitch, 3 = knot, 4 = other (e.g. burn)), size = length of hole in cm, and location = distance for the midpoint of the hole to the bottom of the net in centimeters (note a value of 180 = roof of net)).
(TXT)

S3 Table Net Holes Less than 0.5 cm Source Data. Columns are as follows: NetID = unique Net ID, DamageID = unique ID for point of net damage,Top of net = indicator for whether the hole was on the roof of the net (1 = yes).
(TXT)
