# Correction: Bed Net Durability Assessments: Exploring a Composite Measure of Net Damage

**DOI:** 10.1371/journal.pone.0133105

**Published:** 2015-07-14

**Authors:** Jodi L. Vanden Eng, Adeline Chan, Ana Paula Abílio, Adam Wolkon, Gabriel Ponce de Leon, John Gimnig, Juliette Morgan

Panel D in Fig 2 is incorrect. Please see the corrected Fig 2 here.

**Fig 2:**
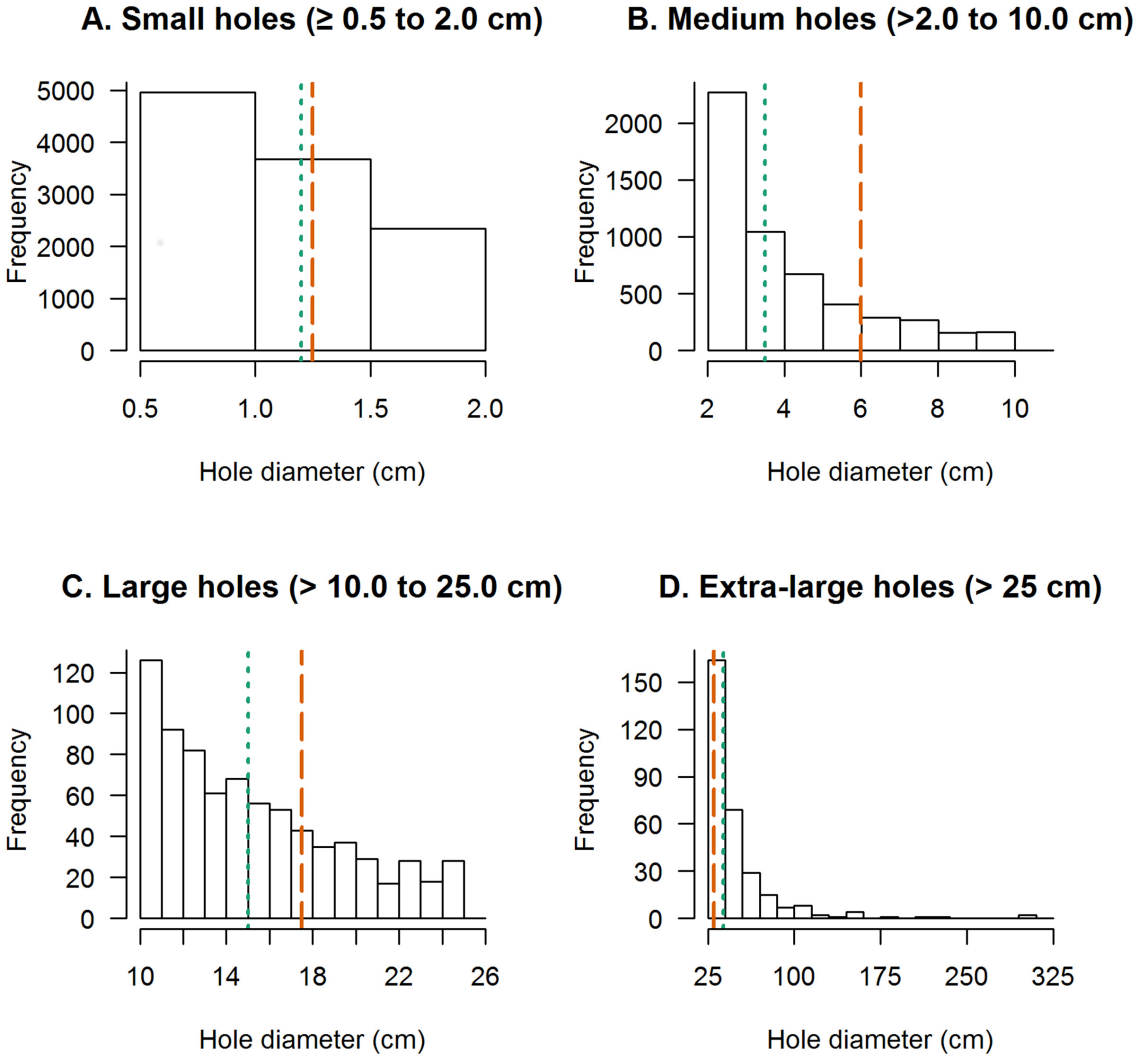
Histogram of measured hole diameters for each assigned WHOPES hole category. The midpoint hole diameter used for the WHOPES category weight is indicated by an orange dashed vertical line. The median hole diameter in each category is indicated by a green dotted vertical line.
